# Early Diagnosis of Delirium in Palliative Care Patients Decreases Mortality and Necessity of Palliative Sedation: Results of a Prospective Observational Study

**DOI:** 10.7759/cureus.25706

**Published:** 2022-06-07

**Authors:** Matteo Beretta, Sara Uggeri, Claudia Santucci, Matteo Cattaneo, Daniela Ermolli, Cristiana Gerosa, Martina Ornaghi, Alessandra Roccasalva, Paola Santambrogio, Giustino Varrassi, Oscar Corli

**Affiliations:** 1 Hospice and Palliative Care, Azienda Socio-Sanitaria e Territoriale (ASST) Brianza, Vimercate, ITA; 2 Oncology and Palliative Care, Mario Negri IRCCS, Milano, ITA; 3 Oncology and Clinical Research, Mario Negri IRCCS, Milano, ITA; 4 Palliative Care, Azienda Socio-Sanitaria e Territoriale (ASST) Brianza, Vimercate, ITA; 5 Palliative Care, Hospice "Fondazione Mantovani", Cologno Monzes, ITA; 6 Research and Education, Paolo Procacci Foundation, Rome, ITA; 7 Research, Mario Negri Institute, Milano, ITA

**Keywords:** mortality, end of life, palliative care, clinical outcome, early diagnosis, delirium

## Abstract

Introduction: Delirium in end-of-life patients is reported to be between 13% and 42% and up to 80% in the terminal phase. It is a serious clinical situation, often a cause of death due to the frequent ineffectiveness of treatments. This study aimed to assess whether and how much precocity of diagnosis, hitherto little considered, could affect the outcomes and prognosis of delirium in palliative care settings.

Methods: Patients consecutively admitted to a palliative care unit (PCU) between October 2018 and December 2019, cared for both in hospice and home programs, were analyzed. All patients were subjected to a careful procedure aimed at recognizing the onset of delirium. The first step was the detection of prodromal "sentinel" symptoms related to incoming delirium. PCU staff and family members/caregivers were trained to observe the patients and immediately identify the appearance of even one symptom. The final diagnosis was performed with the 4AT (4 A’s test). Patients were then included in the categories of "early" or "slow" diagnosis (cut-off: four hours) depending on the time between sentinel symptom observation and the final diagnosis of delirium.

Results: Among 503 admitted patients, 95 developed delirium. Confusion was the most frequent sentinel symptom (49.5%). The early diagnosis was more frequent in hospice than in home care (p-value<0.0001). Delirium was positively resolved in 43 patients, of which 25 with an early diagnosis (p-value=0.038). Time to resolution was shorter in the case of early diagnosis (7.1 vs. 13.7 hours in hospice patients; p-value=0.018). Palliative sedation was performed on 25 patients, but only 8 of them had an early diagnosis.

Conclusion: Time of diagnosis was important in determining the clinical outcomes of patients in charge of PCU who experienced delirium. The early diagnosis reduced both mortality and the necessity of palliative sedation.

## Introduction

According to the fifth edition of the Diagnostic and Statistical Manual of Mental Disorders (DSM-5) [1], the definition of delirium is a disturbance in attention and awareness that develops acutely and tends to fluctuate. The American Psychiatric Association also specifies that delirium is characterized by inattention, disturbance of consciousness, and disorganized thinking [2]. Its occurrence is due to various pathologies, sometimes simultaneously present, such as dementia, organ functional deficits, multimorbidity, and psychiatric disorders treated with drugs that act on the central nervous system, particularly narcotics, hypnotics (such as benzodiazepines), and anticholinergics [2-7]. In addition, older age and hospitalization facilitate the onset of delirium [8-10]. Its prevalence in patients with advanced cancer admitted to hospitals has been estimated at 28% to 48% [11]. The presence of delirium in patients close to the end of life and cared for in palliative services is reported to be between 13% and 42% at the moment of admission to palliative care units (PCU) and tends to increase to up to 80% in the last days/hours of life [12]. Delirium in terminal patients is a very serious clinical situation that is often the cause of death [13,14]. Its severity can also depend on treatments that are frequently ineffective.

Antipsychotics (especially haloperidol) are used as a first-line therapeutic approach in both hyperactive and hypoactive or mixed delirium, although their efficacy is controversial. A meta-analysis [15] found that antipsychotics had no significant effects on delirium duration, severity, and mortality in patients with advanced illness. A recent study [16] made it clear underlining that delirium tended to worsen or poorly respond in palliative care patients.

All these premises seem to give little hope to face positively delirium in advanced patients in charge of palliative care. Therefore, we decided to continue a previously started line of research.

A prospective, single-center, cohort study was conducted in 2018-2019 at the specialist PCU of Giussano, ASST Brianza (MB), Italy. Our first publication on the whole cohort of 503 patients aimed to identify relevant clinical factors that could be related to the risk of delirium onset [17], and we found that the setting of care, presence of breathlessness, and administration of psychoactive drugs, particularly haloperidol, were significantly associated with the risk of developing delirium.

In this study, we focused on some aspects concerning the clinical outcomes of delirium. Other factors, hitherto little considered, could weigh in on controlling the clinical outcomes of delirium.

This study, analyzing the 95 patients from the original cohort who experienced delirium, aimed to measure if and how much precocious diagnosis could affect the clinical characteristics, the outcomes, and the prognosis of delirium in palliative care settings.

## Materials and methods

Study design and participants

As already described [17], a total of 503 patients aged 18 or more were enrolled between October 2018 and December 2019 and followed till the date of delirium resolution, death, transfer outside the PCU, patient withdrawal, or end of follow-up (October 28, 2020).

The Ethics Committee of the ASST of Vimercate (Italy) approved this study on June 18, 2018 (project no. 2824). Written informed consent for participation in the study and processing of personal data was collected from all recruited patients before any study-related activity was carried out.

Briefly, the patients were cared for both in hospice and through home care programs, employing a unique staff in both the settings used to apply internal clinical protocols, thus ensuring homogeneity of care. At the moment of admission, patients with a chronic progressive disease and able to comprehend and speak Italian were consecutively enrolled. Patients with a state of coma, diagnosis of psychiatric pathology, dementia, or substance abuse and/or dependence, current or last for at least three months, were excluded. Moreover, patients with delirium already in progress at the time of admission were excluded. Main baseline characteristics of the patients, including age, sex, education, marital status, primary pathology, Karnofsky Performance Status (KPS), and the setting of care, were recorded. The information about the setting of care was also collected during the delirium episode since patients can move during the follow-up.

Delirium recognition occurs through a specific procedure. The first step was the detection of prodromal symptoms related to incoming delirium. We called them "sentinel" symptoms that consisted of a lack of attention, confusion, drowsiness, poor spatial or temporal orientation, memory deficits, language difficulty, and sensorial alterations [16,18-22]. To identify the sentinel symptoms, PCU staff were trained to observe the patients and to immediately identify the appearance of even one of these indicators.

For patients being cared for at home, one or more family members/caregivers were trained by the medical-nursing staff to recognize the first manifestation of delirium. In particular, it was explained to pay attention to the appearance of fluctuations in the state of orientation, confusing states, changes of attitude, as well as relationships with the family caregivers. At the first appearance of at least one of these signals, caregivers were required to immediately contact the doctors and/or nurses of the PCU. The final diagnosis of delirium was carried out using the validated Italian version of the 4 A’s Test (4AT) [23], which has been proven to have good diagnostic accuracy [24-26]. The 4AT test includes questions to investigate the patient’s state of supervision, orientation, attention, and the presence of acute change or fluctuating courses. The final score ranges from 0 to 12, and a 4AT score ≥4 indicates a delirium in progress. Both the medical and nursing staff of the PCU could carry out the diagnosis with 4AT. Depending on the interval of time between the first suspicion, due to the appearance of at least one sentinel symptom, and the definitive diagnosis, patients were included in two categories, defined as an early or slow diagnosis. More precisely, we decided to divide this variable using the median time (i.e., four hours) as a cut-off that separated the diagnosis categories. In patients with delirium, the severity of symptoms was assessed according to the Delirium-O-Meter (DOM) [27] and the psychometric characteristics of the Delirium Motor Subtype Scale (DMSS) [28]. Both DOM and DMSS are reliable and valid tests based on literature data [27,29-31]. The treatments chosen by the PCU doctors were collected in terms of administered drugs and range of doses. Finally, the type and time of the resolution of the crisis were recorded.

Statistical analysis

Descriptive statistics were used to summarize the patients’ demographic and clinical characteristics. The care setting, mean DOM, and psychometric subtype were compared between patients with slow or early diagnosis to understand which factors were significantly related to an early diagnosis. Differences between patients with and without an early diagnosis were analyzed using the t-test and chi-square test, respectively, for continuous and categorical variables. For statistical analyses, we used the software SAS version 9.4 (SAS Institute Inc., Cary, NC, USA).

## Results

Among 503 patients admitted, 95 developed delirium during the study. Table 1 shows the main baseline characteristics of these 95 patients. Fifty-eight patients were male (61.1%), and the mean age was 78.2 years.

**Table 1:** Main baseline characteristics among 95 patients admitted to palliative care who experienced delirium. SD: standard deviation, KPS: Karnofsy Performance Status.

Characteristics	N (%)
Sex, male	58 (61.1)
Age, years	
≤70	21 (22.1)
71-80	30 (31.6)
>80	44 (46.3)
Mean (SD)	78.2 (11.0)
Education	
Primary school or less	50 (52.6)
Middle school	24 (25.3)
High school or University degree	21 (22.1)
Marital status	
Single	7 (7.4)
Married	52 (54.7)
Widow/widower	33 (34.7)
Separate	2 (2.11)
Cohabiting	1 (1.1)
Primary disease	
Cancer	82 (86.3)
Other diseases	13 (13.7)
Respiratory	0 (0)
Heart	2 (2.1)
Liver	3 (3.2)
Vascular	0 (0)
Kidney	2 (2.1)
Other	6 (6.3)
Setting of care at enrolment	
Home care	47 (49.5)
Hospice	48 (50.5)
KPS	
≤30	32 (33.7)
40	30 (31.6)
≥50	33 (34.7)

Only 21 (22.1%) patients had a high level of education, and 52 (54.7%) were married. Cancer was the primary pathology in 82 (86.3%) patients, and KPS was ≤30 for 32 (33.7%) patients. The setting of care at enrollment was a hospice for 48 (50.5%) patients. Table 2 includes the sentinel symptoms found among 95 patients.

**Table 2:** The number and frequency of sentinel symptoms perceived either as first symptom and during the delirium among 95 patients admitted to palliative care who experienced delirium.

Symptoms perceived	First symptom N (%)	During the delirium N (%)
Lack of attention	8 (8.4)	48 (50.5)
Confusion	47 (49.5)	66 (69.5)
Drowsiness	17 (17.9)	33 (34.7)
Poor spatial orientation	8 (8.4)	35 (36.8)
Poor temporal orientation	1 (1.1)	42 (44.2)
Memory deficits	1 (1.1)	15 (15.8)
Language deficits	3 (3.2)	21 (22.1)
Alterations to the five senses	10 (10.5)	19 (20.0)

Confusion was the most frequent first indicator (49.5%), followed by drowsiness (17.9%), alterations to the five senses (10.5%), lack of attention (8.4%), and poor spatial orientation (8.4%). Caregivers who caught the first sentinel were 57.9% healthcare professionals, 17.9% spouses, 14.7% sons or daughters, 3.2% care workers, and 6.3% others (data not shown). The diagnosis, achieved by 4AT, was carried out in 44.2% of cases by doctors, and the mean score of 4AT was 8.5 (SD 2.5) (data not shown). Table 3 reports the setting of care for delirium, the clinical severity, and the psychometric subtypes of delirium in relation to the time of diagnosis.

**Table 3:** The setting of care, time of diagnosis, clinical severity of delirium (according to the delirium-O-meter), and psychometric subtype in relation to diagnosis time among 95 patients admitted to palliative care who experienced delirium. SD: standard deviation. ^a^Differences between early and slow diagnosis were tested using chi-square tests or t-tests.

	Early diagnosis (<4 h)	Slow diagnosis (≥4 h)	p-value^a^
	N (%)	N (%)	
All patients	49	46	
Setting of care			
Homecare	3 (6.1)	25 (54.4)	
Hospice	46 (93.9)	21 (45.7)	<0.0001
DOM			
Mean (SD)	16.2 (6.1)	18.3 (6.4)	0.118
Psychometric subtype			
Hyperactive	18 (36.7)	9 (19.6)	
Hypoactive	16 (32.7)	22 (47.8)	
Mixed	12 (24.5)	12 (26.1)	
Missing	3 (6.5)	3 (6.5)	0.281

Timing differences are mainly determined by the setting of care, with early diagnosis much more frequent in hospice than in home care (p-value 0.0001), while the severity of the clinical picture, measured by DOM, and motor characteristics of delirium did not show significant differences between early and slow diagnosis. In Table 4, the therapeutic prescriptions made in coincidence with the diagnosis of delirium are reported.

**Table 4:** Pharmacological treatments for delirium among 95 patients admitted to Palliative Care who experienced delirium. IQR: interquartile range.

Drugs	N (%)	Median dose and IQR (mg)
Haloperidol	49 (51.6)	2.0 (2.0–4.0)
Citalopram	1 (1.1)	20.0
Midazolam	34 (35.8)	5.0 (5.0–15.0)
Morphine	34 (35.8)	20.0 (10.0–50.0)
Scopolamine	17 (17.9)	60.0 (20.0–60.0)
Quetiapine	2 (2.1)	25.0
Fentanyl	1 (1.1)	12.0
Zolpidem	1 (1.1)	10.0

Among drugs, haloperidol (51.6%), midazolam (35.8%), morphine (35.8%), and scopolamine (17.9%) prevailed. These therapeutic choices are here simply described as not falling within the specific objectives of the study.

The delirium outcomes after the treatments consisted of a positive resolution for 43 (45.3%) patients and death after palliative sedation for 25 (26.3%) patients. Twenty-seven patients who naturally died during the delirium episodes (28.4%) were separately considered. When comparing delirium outcomes in relation to the time of diagnosis, we found that positive resolution was significantly present in 75.8% of patients with an early diagnosis and 51.4% of patients with a slow diagnosis (p-value = 0.038; Table 5). Among 43 patients who positively resolved the delirium episode, we investigated the time to resolution in the different settings and in correlation with the time of diagnosis (Table 5).

**Table 5:** Resolution of delirium and time to resolution among 43 patients who resolved in relation to diagnosis time, among 95 patients admitted to palliative care. SD: standard deviation. ^a^Difference between early and slow diagnosis was tested using chi-square tests or t-tests.

	Early diagnosis (<4 h)	Slow diagnosis (≥4 h)	p-value^a^
	N (%)	N (%)	
Number of patients	33	35	
Resolution			
Palliative sedation (n=25), N (%)	8 (24.2)	17 (48.6)	0.038
Delirium resolved (n=43), N (%)	25 (75.8)	18 (51.4)	
Time to resolution			
At home, mean (SD)	8.9 (9.2)	13.5 (10.2)	0.816
In hospice, mean (SD)	7.1 (5.0)	13.7 (11.2)	0.018

In each case, the time to resolution was shorter when the diagnosis was early. The difference was not significant in the patients followed at home, but was significant (p-value=0.018) in the patients followed in hospice.

## Discussion

Time of diagnosis is important in determining the clinical outcomes of patients in charge of palliative care who experience delirium. The effects of an early diagnosis consist of reduced mortality and the necessity of palliative sedation.

We studied a population of 95 patients (77.9% aged 70 years or more) in severe clinical conditions (65.3% with KPS ≤40), all suffering from delirium. Both these conditions normally have a negative prognosis. In all the patients admitted to PCU, we started investigating the possible presence of sentinel symptoms through the active collaboration of caregivers or health professionals. The reported found sentinel symptoms were followed by the execution of 4AT to confirm the diagnosis, and immediately after, by the administration of therapy. The overall time of diagnosis allowed us to divide the whole sample into two time-category subgroups: the first when the time was less than four hours and the second when it was more than four hours. We performed all the statistical analyses, comparing the results in relation to these two categories. First, we observed the importance of the palliative care setting. Our previous publication found that hospice care is associated with a higher risk of developing delirium (HR=2.28, 95% CI 1.45-3.60) as compared to home care [17], while this study, which is not focused on risk factors for delirium but on the role that may have the time to diagnosis on delirium clinical outcomes, highlights a different aspect concerning the setting of care. Among 49 patients with early diagnosis, 46 were managed in hospice and only 3 in a home care program. It was obvious that patients in hospice were continuously under observation, allowing the entire diagnostic path to be tracked in real-time. This aspect, however, reinforced the idea that reaction time was important, and the place of care was a conditioning factor. Second, the relationships between the time of diagnosis, the severity of delirium, and psychometric subtypes had no significant differences. This is important, considering that delirium, especially hypoactive delirium, can carry a worse prognosis. The main objective was to estimate the relationship between time of diagnosis and clinical outcomes. In general, three solutions to the delirium episodes occurred: the first, positive, consisting of the resolution of the episode and return to a normal condition; the other two, negative, related to the death of the patient, occurred naturally or following palliative sedation. In comparison with another study [32] in which 56% of palliative care patients died within 14 days from the beginning of delirium, in our study only 28.4% naturally died. Natural death was an expected, short-term event. Delirium was a further complication that arose in an already compromised clinical picture. In the 27 patients who died naturally, death was the consequence of the organ dysfunctions, symptoms, and infectious complications that affect a terminally ill patient, plus the presence of delirium. This situation was expected and not particularly different from what commonly happens in the clinic experience [33]. In presenting the results, we considered natural death as a spontaneous event, not responsive to therapy and not indicated for palliative sedation. We compared, instead, the two outcomes related to specific treatments carried out: the resolution of the crisis due to a good response to delirium treatments; and death following sedation as a response to a therapy aimed only to relieve the unmanageable patient’s suffering. Positive resolution occurred in 25, out of 33 (75.8%), patients with an early diagnosis and in 18, out of 35 (51.4%), patients after a slow diagnosis. In parallel, sedation was needed in 24.2% of early diagnoses and 48.6% of the slow ones. Comparing these two situations, in which delirium treatment plays a substantial part, the relationship with time of diagnosis had a decisive role.

The management of refractory delirium through palliative sedation to reduce patient distress is considered an appropriate intervention [34,35], ethically and legally accepted [13, 36]. In our study, palliative sedation was applied to 25 patients, of which 8 were in the group of early diagnosis and 17 were in the group of delayed diagnosis.

Trying to interpret the results, the collected data were characterized by some constants, such as the advanced age of the patients, severe clinical conditions, being in charge of palliative care, the occurrence of delirium, and the administration of treatments usually given for this pathology [20]. In all cases, the only clinical variant capable of strongly affecting the outcomes of delirium episodes was the time of diagnosis.

A separate consideration deserves the introduction in this study of an original diagnostic procedure. The literature reports that delirium is often undetected or misdiagnosed [24,37-40]. The use of the early detection method, based on the use of sentinel symptoms, allowed the identification of 95 cases of delirium in 503 patients. These cases accounted for 100% of the diagnoses, as no other cases emerged during the whole study follow-up.

The main limitation of this study could be its monocentric implementation, which precludes comparison and generalizability of results. It has also led to some advantages, such as accurate and uniform training of the health professionals and caregivers involved in the clinical assessments and data collection. There are also some strengths in this study, such as the originality of the primary objective, the active involvement of family members and caregivers in the evaluation procedures, and the achieved results that can everywhere induce the early diagnosis of delirium.

## Conclusions

This study identified the importance of a rapid diagnostic procedure for delirium in palliative care patients as a key aspect in improving their prognosis. Additional data and a future active sharing experience with other PCUs would be worthwhile to confirm these findings and their usefulness in clinical practice and to compare the treatments used in this setting. From now on, clinicians in the PCUs must be alerted to the early recognition and treatment of patients with incoming delirium. Their attention can give them more days ahead and a more lucid and aware preparation for the end of life.
